# Unveiling the role of YARS1 in bladder cancer: A prognostic biomarker and therapeutic target

**DOI:** 10.1111/jcmm.18213

**Published:** 2024-03-20

**Authors:** YaXuan Wang, Jinfeng Wang, Lu Zhang, JiaXing He, Bo Ji, JianShe Wang, BeiChen Ding, MingHua Ren

**Affiliations:** ^1^ Department of Urology The First Affiliated Hospital of Harbin Medical University Harbin China

**Keywords:** ceRNA, DNA methylation, immune infiltration, prognosis, senescence

## Abstract

YARS is responsible for catalysing the binding of tyrosine to its cognate tRNA and plays a crucial role in basic biosynthesis. However, its biological functions in bladder cancer remains to be proven. We analysed variations in YARS1 expression and survival in bladder cancer using multiple data sets, including TCGA‐BLCA, GSE13507 and bladder cancer‐specific tissue microarrays. Furthermore, we explored the biological functions of YARS1 using transcriptome data. Our findings revealed a noteworthy correlation between YARS1 and immune infiltration in bladder cancer, as determined using the XCELL algorithm and single‐cell analysis. In addition, we employed the TIDE algorithm to evaluate the responsiveness of different cohorts to immune checkpoint therapy. We investigated the regulatory associations between YARS1 and various aspects of bladder cancer, including senescence, ferroptosis and stemness. Finally, we established a ceRNA network that is directly linked to the overall prognosis, YARS1 can serve as a prognostic biomarker for bladder cancer; its interaction with MYC has implications for bladder cancer cell senescence, ferroptosis and stemness. Moreover, the identified ceRNA network has potential as a therapeutic target in bladder cancer.

## 1 INTRODUCTION

Bladder cancer is a prevalent form of malignant tumour found in males and presents a significant danger owing to its high occurrence and mortality rates. Various treatment options, including surgery, radiotherapy and immunotherapy, are available for managing bladder cancer.^1^ However, the prognosis of patients with bladder cancer remains unsatisfactory due to recurrence, metastasis and drug resistance.^2^ The current standard of care is most effective for individuals with an early‐stage diagnosis of bladder cancer, but patients diagnosed at an advanced stage have limited treatment options. Recognizing the significance of genomic alterations in the development of bladder cancer,^3^ it is of utmost importance to extensively investigate the underlying mechanisms of this disease and identify new therapeutic targets.

Housekeeping proteins of the aminoacyl‐tRNA synthetase (aaRS) family are widely found in all forms of life. Their primary roles involve catalysing the binding of amino acids to tRNAs and facilitating the translation of genetic information from nucleic acids to amino acids. Consequently, they play vital roles in protein synthesis.^4^ Among the various types of aaRS, YARS (tyrosyl‐tRNA synthetase, also referred to as TyrRS or YRS) specifically aids in the binding of tyrosine to its corresponding tRNA.^5^ Recent research has highlighted that aaRS also engages in multiple pathway networks by forming the multi‐tRNA synthetase complex (MSC), which opens new avenues for the therapeutic targeting of aminoacyl‐tRNA synthetases in immune disorders, rare diseases and even cancers.^6^, ^7^ Furthermore, studies have identified YARS as an oncogenic protein that promotes the progression of gastric cancer by activating the PI3K‐Akt signalling pathway.^8^ However, there is currently a dearth of research exploring the biological function of YARS1 in bladder cancer and its potential as a therapeutic target.

This study aimed to explore the expression and prognostic significance of YARS1 in bladder cancer using The Cancer Genome Atlas (TCGA) BLCA and GSE13507 data sets. Additionally, we investigated the potential role of YARS1 in bladder cancer using gene enrichment analyses. Our findings revealed that YARS1 interacts with the well‐known oncogene MYC, and together they play crucial roles in regulating ferroptosis, senescence and stemness in bladder cancer cells. Furthermore, we constructed a ceRNA network involving YARS1. Overall, our results suggest that YARS1 could serve as a valuable prognostic biomarker for bladder cancer and may have a significant impact on various biological functions of bladder cancer cells.

## 2 MATERIALS AND METHODS

### 2.1 Data sets and patient samples

This study utilized the bladder cancer data set from TCGA and Gene Expression Omnibus (GEO) databases. The TCGA‐BLCA data set comprises 406 bladder cancer samples and 19 normal bladder tissue samples. The GSE13507 data set from the GEO database included 165 primary bladder cancer samples and nine normal bladder tissue samples. Relevant clinical information was collected for both data sets. Bladder cancer tissue microarrays were procured from Shanghai Outdo Biotech Company, and 49 patients were included in the study. Samples with unclear TNM stages or those lost to follow‐up were excluded, resulting in the final inclusion of 46 bladder cancer samples and 29 normal bladder tissue samples. Surgical procedures were conducted between May 2007 and November 2011, with follow‐up evaluations performed on March 2014, covering a period ranging from 2.3 to 7 years. Ethical approval was obtained before the start of the study.

### 2.2 Immunohistochemical analysis of YARS1 expression in bladder cancer tissue microarrays and prognostic relevance

Tissue microarrays were subjected to a series of processing steps. Initially, they were placed in an oven at 85°C for 10 min. They were then soaked in xylene for 15 min and subjected to hydration using a gradient of ethanol concentrations of 100%, 95%, 80% and 70%. Subsequently, the chips were treated with a citric acid solution in an autoclave to facilitate antigen repair. Once the chips were cooled, they were rinsed with PBS and exposed to hydrogen peroxide for 20 min. Following this step, the YARS1 antibody (ab150429) was added, with an incubation period of 2 h at room temperature. The above procedure was considered complete once the tissue microarrays were rinsed three times with PBS and then subjected to a 20‐min incubation with an immunohistochemical secondary antibody at room temperature. After an additional rinsing with PBS three times, the microarrays were stained with DAB, followed by staining with haematoxylin. Subsequently, the microarrays were dehydrated using an ethanol gradient (70%, 80%, 90% and 100%). Finally, they were immersed in xylene for 8 min, after which the microarrays were blocked, marking the culmination of the aforementioned processes. The immunostaining intensity scores ranged from 0 to 3, with 0, 1, 2 and 3 representing no reaction, weak reaction, moderate reaction and strong reaction respectively. Subsequently, the scales were assigned scores according to the proportion of positive staining observed: scores of 1, 2, 3 and 4 indicated ranges of 0%–25%, 26%–50%, 51%–75%, and 76%–100% respectively. The final scores were calculated by multiplying the strength scores with the proportional scores. The results were interpreted as follows: Scores ranging from 0 to 5 indicated low expression, whereas scores ranging from 6 to 12 indicated high expression.

### 2.3 Gene enrichment analysis

The Limma package available in R software (version: 3.40.2) was used to investigate the differential expression of the mRNAs. To screen for mRNA differential expression, a threshold was set as ‘*p* < 0.05 and Log2 (Fold Change) >2 or Log2 (Fold Change) <−2’. To gain a deeper comprehension of YARS1's oncogenic role, the ClusterProfiler package in R was used to analyse possible Gene Ontology (GO) functions and enrich the Kyoto Encyclopedia of Genes and Genomes (KEGG) pathway. The ClusterProfiler package was used to examine potential features of Gene Set Enrichment Analysis (GSEA).^9^

### 2.4 Immune infiltration analysis

To ensure a reliable evaluation of the immune score results, we employed immunedeconv, an R software package.^10^ Each algorithm was thoroughly tested, and each offered unique advantages. In this study, we selected the XCELL method because it assesses a wider range of immune cells.^11^ Furthermore, we utilized the LASSO algorithm to identify immune cells with prognostic relevance. All the above analysis methods and R packages were implemented using the R Foundation for Statistical Computing (2020) version 4.0.3.

### 2.5 DNA methylation analysis

Data on the differences in methylation levels of YARS1 between bladder cancer and normal bladder tissues as well as across various stages were obtained using the UALCAN database.^12^ Additionally, the SMARP database provided information on changes in expression and correlation analysis of methylation probes that target YARS1 in bladder cancer.^13^

### 2.6 Stemness score of tumour cells

We used the OCLR algorithm to determine mRNAsi, a novel metric devised by Malta et al.^14^ The gene expression profiles comprised a comprehensive collection of 11,774 genes. Spearman's correlation analysis was used to examine the RNA expression data. Subsequently, we normalized the resulting value within the range of [0, 1] by subtracting the minimum value and dividing it by the maximum value. This transformation was performed to represent the dryness index accurately. The analysis methods and R package were implemented using the R Foundation for Statistical Computing (2020) version 4.0.3.

### 2.7 CeRNA network analysis

YARS1‐related miRNAs were analysed by ENCORI and TarBase v.8 databases.^15^, ^16^ miRNA‐related circRNAs were analysed using the ENCORI database.

### 2.8 Statistical analysis

YARS1 expression in bladder cancer and normal bladder tissues was determined by the Wilcoxon rank‐sum test. Prognostic analysis was performed using the log‐rank test. Statistical significance was defined as *p* < 0.05.

## 3 RESULTS

### 3.1 YARS1 is highly expressed in bladder cancer

**FIGURE 1:**
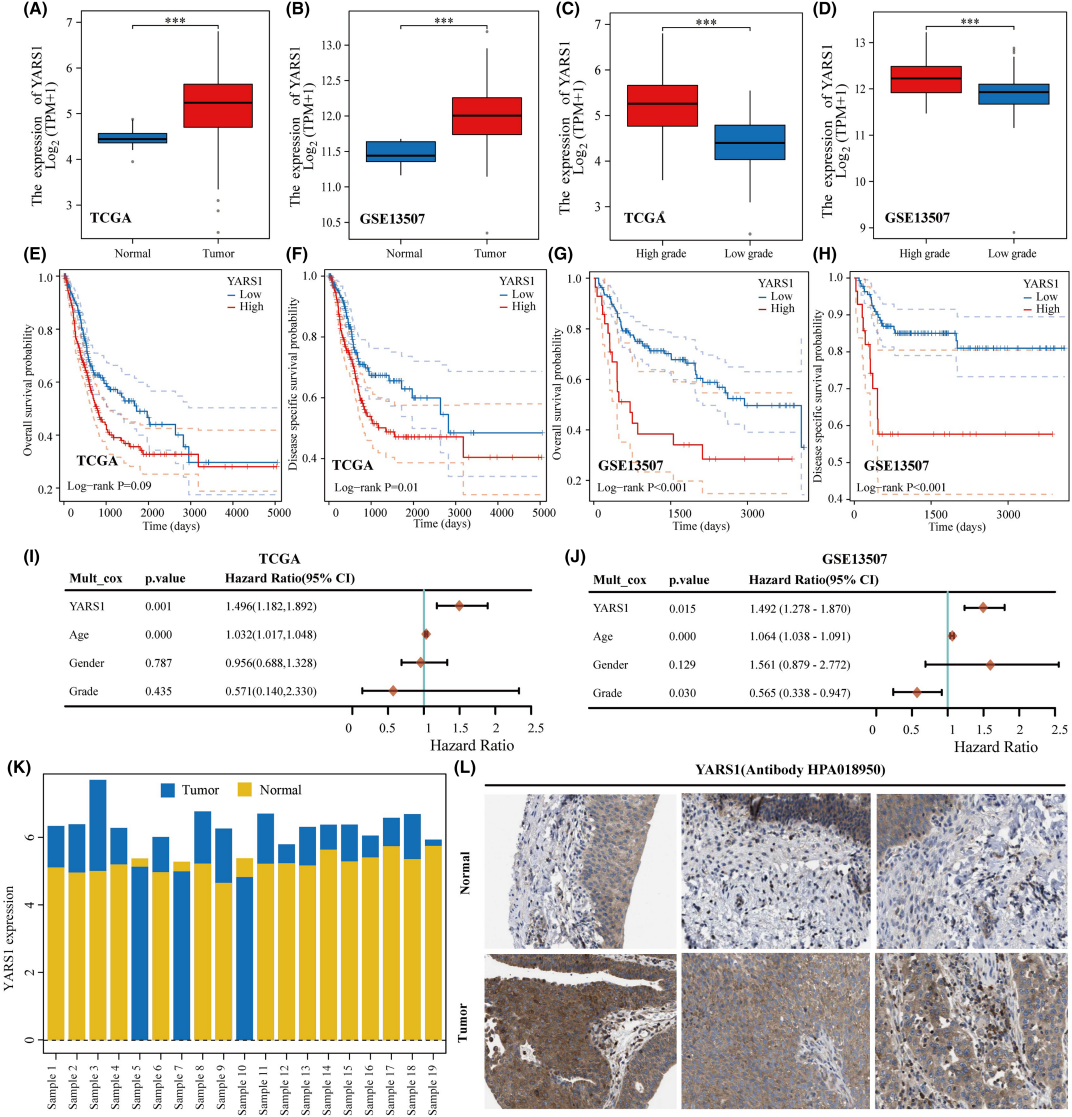
Poor prognosis is observed in patients exhibiting high expression of YARS1. (A, B) Expression of YARS1 in the TCGA and GSE13507 databases compared between bladder cancer and normal tissues. (C, D) Analysis of YARS1 expression in high‐grade and low‐grade bladder cancer using the TCGA and GSE13507 databases. (E–H) Evaluation of the correlation between YARS1 expression and overall and disease‐specific survival in TCGA‐BLCA and GSE13507 patients. (I, J) Assessment of the prognostic significance of YARS1 in bladder cancer through multifactorial Cox regression analysis. (K) Bar graph depicting YARS1 expression in matched bladder cancer samples from the TCGA‐BLCA data set. (L) Comparative analysis of YARS1 protein expression between bladder cancer tissues and normal bladder tissues. ***p < .001.

We investigated YARS1 mRNA levels in normal bladder tissues and bladder cancer using two different data sets: TCGA‐BLCA and GSE13507. Our findings revealed a consistent elevation in the expression levels of YARS1 in bladder cancer samples compared to those in normal bladder tissues (Figure 1A, B). In clinical practice, bladder cancer grading is based on the level of malignancy. High‐grade bladder cancers are more malignant than low‐grade cancers. Hence, we compared the expression of YARS1 in high‐ and low‐grade bladder cancers, and our results demonstrated a significant increase in YARS1 expression in high‐grade bladder cancer. This observation suggests a correlation between high YARS1 expression and bladder cancer (Figure 1C, D). To ascertain the prognostic relevance of YARS1, we analysed the TCGA‐BLCA data set and discovered that patients with high expression of YARS1 exhibited poorer overall and disease‐specific survival rates (Figure 1E, F). The prognostic significance of YARS1 was further confirmed using the GSE13507 data set (Figure 1G, H). Furthermore, we performed a multifactorial Cox regression analysis of YARS1, which considered factors, such as patient age, sex and grading factors, from the TCGA‐BLCA data set. The results of this analysis provided additional support for the substantial role of YARS1 in determining the prognosis of bladder cancer (Figure 1I). This was validated using the GSE13507 data set (Figure 1J). The utilization of bar graphs allowed for the visual representation of the disparity in YARS1 expression in paired TCGA‐BLCA samples. Our findings revealed a significant increase in YARS1 expression in 19 paired bladder cancer cases compared to that in paired normal bladder tissues (Figure 1K). We analysed the disparities in YARS1 protein expression between bladder cancer samples and normal bladder tissues using The Human Protein Atlas database (Figure 1L). In summary, our results shed light on the increased expression of YARS1 in bladder cancer and its association with poor patient prognosis.

### 3.2 YARS1 can be used as a prognostic marker for bladder cancer

**FIGURE 2:**
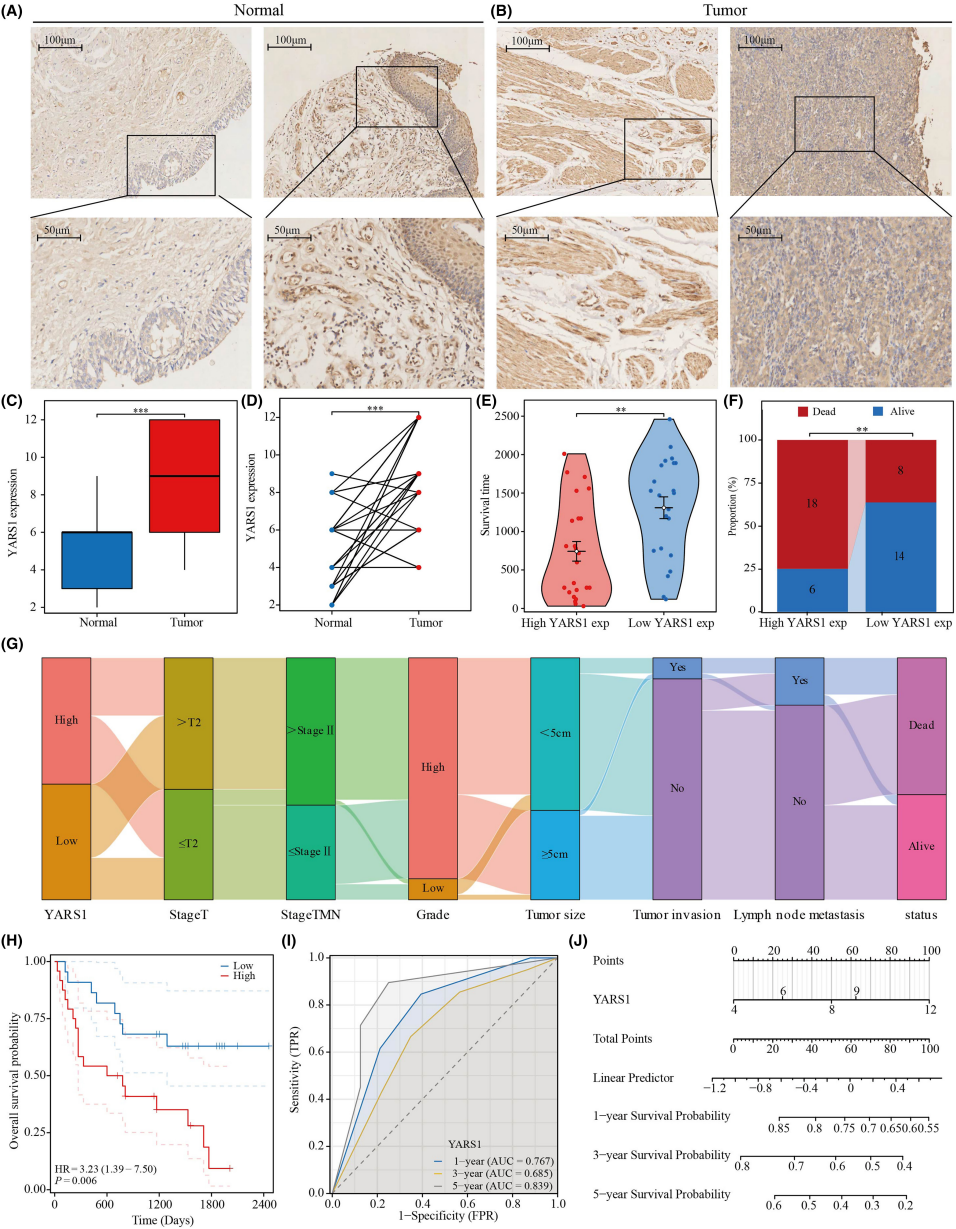
In order to verify the expression of YARS1 in bladder cancer and assess its prognostic significance, tissue microarray analysis was performed. (A, B) The level of YARS1 expression was examined in both bladder cancer and normal tissues using immunohistochemical staining. (C) A comparison was made between YARS1 expression in unmatched bladder cancer and normal bladder tissues. (D) YARS1 expression was evaluated in paired bladder cancer tissues. (E) Prognostic value of YARS1 expression was assessed by analysing the survival time difference between patients in the high‐expression and low‐expression groups. (F) Survival/death ratio of patients in the high‐expression group and low‐expression group of YARS1. (G) Distribution patterns of bladder cancer patients based on stage, grade and other clinical factors. (H) YARS1 expression was subjected to Kaplan–Meier survival analysis. (I, J) Predictive ability of YARS1 expression for the 1‐, 3‐ and 5‐year prognosis of bladder cancer patients was analysed. **p < .01, ***p < .001.

To explore the expression and prognostic significance of YARS1 in bladder cancer, immunohistochemical experiments were conducted on 46 bladder cancer tissues and 29 normal bladder tissues. Our findings are consistent with those of TCGA‐BLCA and GSE13507 data sets. The results indicated a substantial increase in YARS1 expression in bladder cancer tissues compared to that in normal bladder tissues (Figure 2A–C). Moreover, within paired samples, YARS1 expression was significantly higher in bladder cancer tissues than in normal bladder tissues (Figure 2D). Survival analysis revealed that patients with decreased YARS1 expression experienced a significantly prolonged survival time compared to those with elevated YARS1 expression (Figure 2E). Furthermore, the YARS1 low‐expression group exhibited a lower incidence of mortality than the YARS1 high‐expression group (Figure 2F). A Sankey plot (Figure 2G) demonstrates the distribution of bladder cancer samples with respect to high and low levels of YARS1 expression, considering variables, such as tumour size, stage, grade, tumour invasion, lymph node metastasis and patient survival status. Important associations were identified using univariate COX regression analyses (Table 1). These associations involved variables, such as TNM stage, T stage, tumour extravasation, lymph node metastasis and YARS1, and were found to significantly impact the prognosis of bladder cancer patients. Furthermore, multivariate COX regression analysis results indicated that YARS1 has the potential to serve as a prognostic biomarker in patients with bladder cancer. Notably, Figure 2H demonstrated that patients with increased YARS1 expression had a considerably worse prognosis. Additionally, the area under the curve (AUC) and column line graphs presented in Figure 2I, J illustrated that YARS1 expression can predict 1‐, 3‐ and 5‐year survival in patients with bladder cancer. In conclusion, our investigation substantiates the elevated expression of YARS1 in bladder carcinoma and the unfavourable prognosis associated with this elevated expression. This validates the potential use of YARS1 as a predictive biomarker for bladder cancer.

**TABLE 1:** COX regression analysis of the prognostic correlation between YARS1 and BLCA.

Characteristics	Total (*N*)	Univariate analysis		Multivariate analysis	
		Hazard ratio (95% CI)	*p* Value	Hazard ratio (95% CI)	*p* Value
Gender	46				
Male	40	Reference			
Female	6	2.085 (0.768–5.657)	0.149		
Age	46	0.990 (0.948–1.034)	0.656		
Grade	46				
High	42	Reference			
Low	4	1.121 (0.262–4.799)	0.878		
Tumour size	46				
<5 cm	29	Reference			
≥5 cm	17	0.708 (0.313–1.605)	0.409		
Tumour invasion	46				
Yes	4	Reference		Reference	
No	42	0.306 (0.104–0.897)	**0.031**	0.190 (0.026–1.387)	0.101
Lymph node metastasis	46				
Yes	9	Reference		Reference	
No	37	0.396 (0.162–0.964)	**0.041**	2.445 (0.386–15.494)	0.343
Stage‐T	46				
>T2	25	Reference		Reference	
≤T2	21	0.348 (0.150–0.807)	**0.014**	2.472 (0.253–24.121)	0.436
Stage‐TMN	46				
>Stage II	28	Reference		Reference	
≤Stage II	18	0.284 (0.113–0.715)	**0.008**	0.055 (0.004–0.776)	**0.032**
YARS1	46	1.237 (1.064–1.440)	**0.006**	1.489 (1.209–1.834)	**<0.001**

*Note*: The table highlights *p* value <0.05 in bold, indicating significant differences between groups.

### 3.3 Differential analysis based on YARS1

**FIGURE 3:**
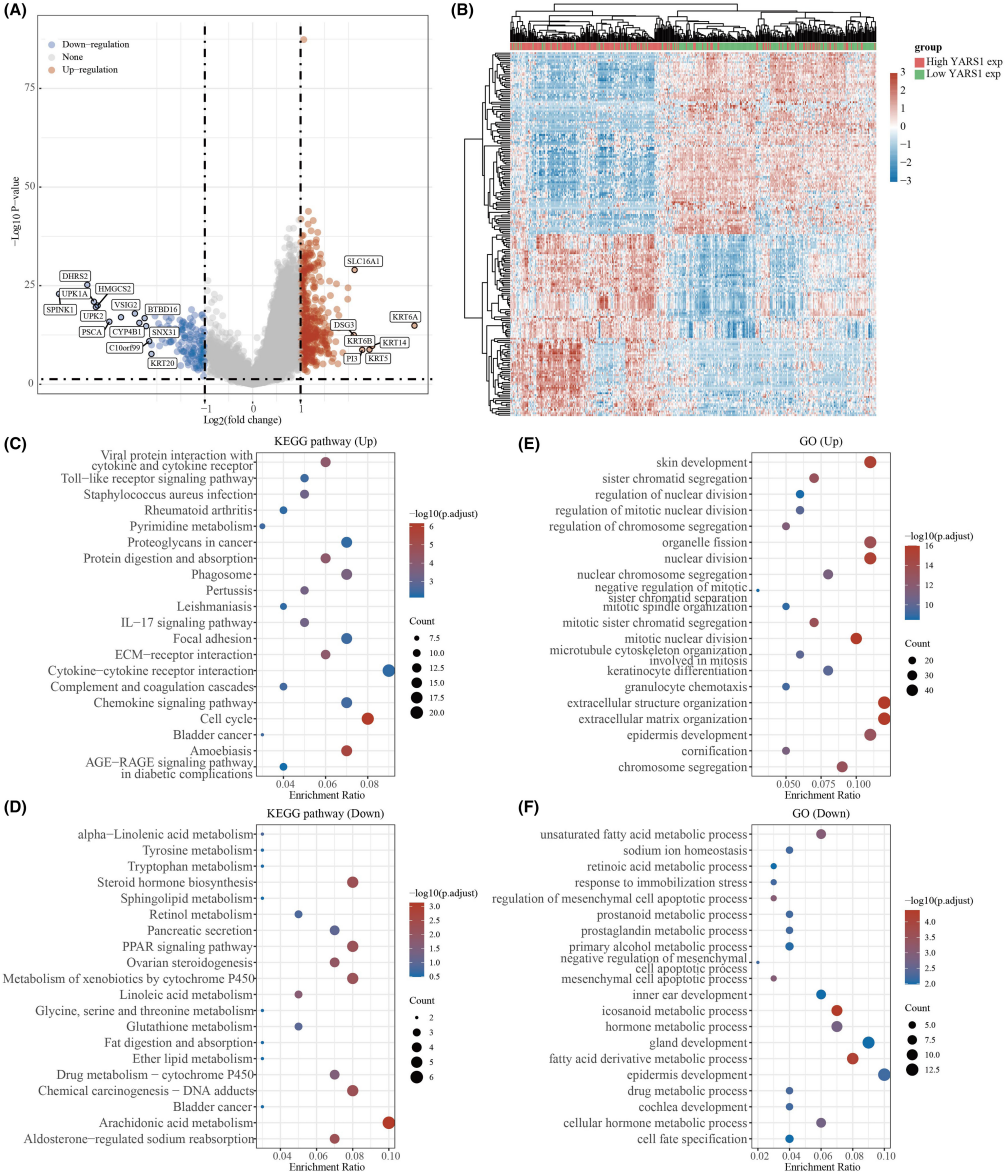
KEGG and GO analyses of potential functions of YARS1. (A) Volcano maps for YARS1 difference analysis. (B) Heat map of YARS1‐related differential gene expression. (C, D) KEGG and GO analysis of YARS1‐related upregulated genes. (E, F) KEGG and GO analysis of YARS1‐related downregulated genes.

In this study, we examined the potential involvement of YARS1 in bladder cancer using differential analysis. To identify differentially expressed genes related to YARS1, we established certain thresholds: *p* < 0.05 and Log2 (Fold Change) >2 or Log2 (Fold Change) <−2. Our findings indicated a significant upregulation of genes, such as SLC16A1, KRT6A, KRT14, KRT5, DSG3, KRT6B and PI3. Conversely, the genes including SPINK1, UPK1A, DHRS2, UPK2, PSCA, VSIG2 and CYP4B1 were significantly downregulated (Figure 3A). To further illustrate the distinction in gene expression between the high YARS1 expression and low YARS1 expression groups, we employed a heat map (Figure 3B). Subsequently, we performed functional enrichment analysis of these differentially expressed genes to validate the potential role of YARS1 in bladder cancer. KEGG analysis demonstrated a significant association between the upregulated genes and various pathways, such as cytokine–cytokine receptor interaction, cell cycle, AGE‐RAGE signalling pathway and chemokine signalling pathway (Figure 3C). Moreover, our GO analysis indicated that the upregulated genes were significantly associated with functions such as extracellular structure organization, extracellular matrix organization, epidermal development and skin development (Figure 3D). Moreover, we observed a significant association between the downregulated genes and various functions through KEGG analysis, including arachidonic acid metabolism, chemical carcinogenesis, DNA adducts, steroid hormone biosynthesis and the PPAR signalling pathway (Figure 3E). Similarly, our GO analysis highlighted the involvement of the downregulated genes in functions related to epidermal development, gland development, fatty acid derivative metabolic processes and icosanoid metabolic processes (Figure 3F). Our results suggest that YARS1 plays an important role in bladder cancer and that the underlying mechanism may be accomplished by regulating the cell cycle, among other pathways.

### 3.4 Gene set enrichment analysis of YARS1 in bladder cancer

**FIGURE 4:**
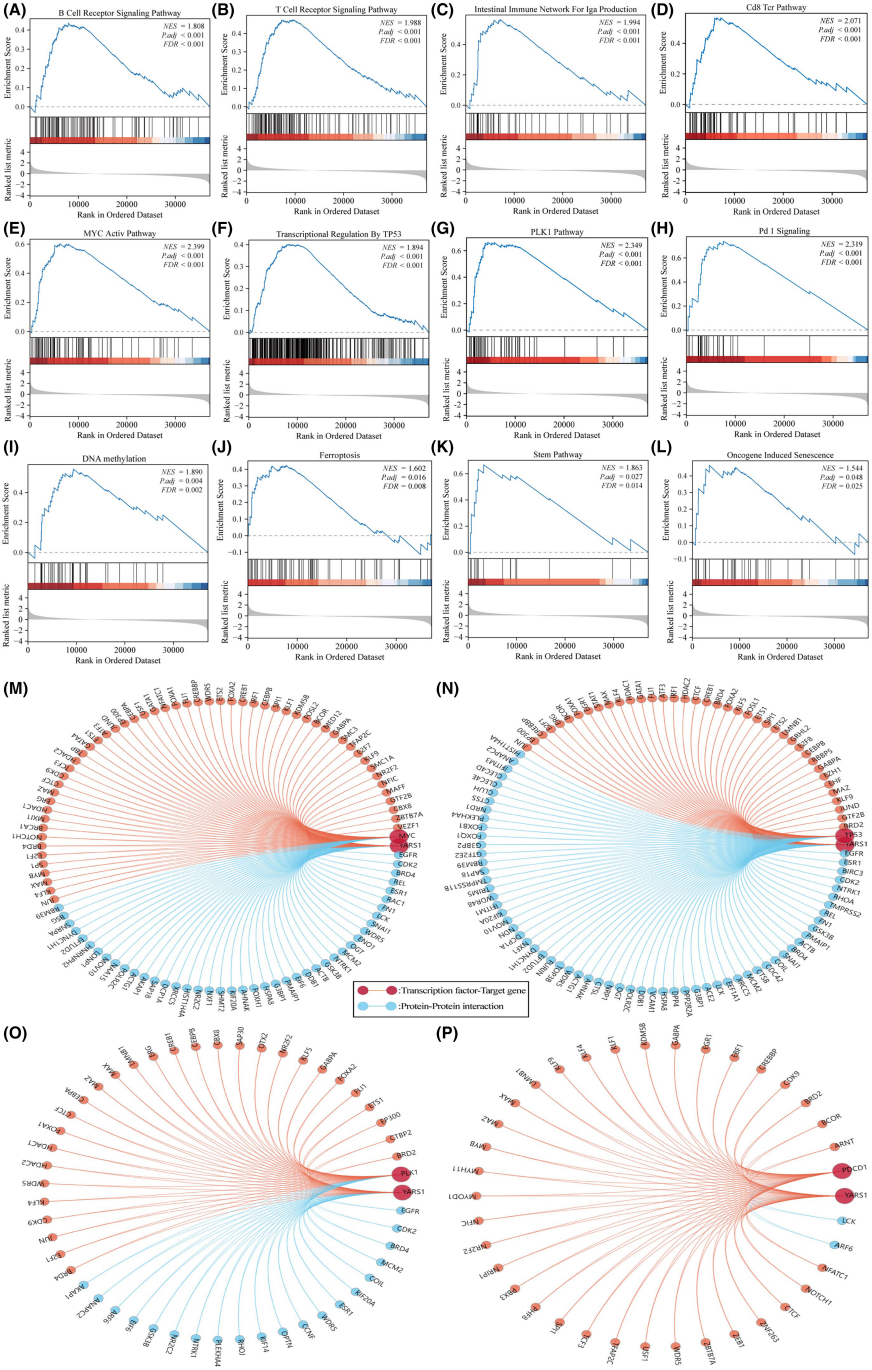
YARS1 plays multiple important functions in bladder cancer. (A) B cell receptor signalling pathway. (B) T cell receptor signalling pathway. (C) Intestinal immune network for IgA production. (D) CD8 Tcr pathway. (E) MYC active pathway. (F) Transcriptional regulation by TP53. (G) PLK1 pathway. (H) PD1 signalling. (I) DNA methylation. (J) Ferroptosis. (K) Stem pathway. (L) Oncogene induced senescence. (M–P) Common reciprocal and target genes between YARS1 and MYC, TP53, PLK1 PDCD1.

General differential analysis (GO and KEGG) typically focuses on comparing gene expression differences between two groups, with an emphasis on significantly upregulated or downregulated genes. However, this approach may overlook genes that are not significantly differentially expressed, but still hold biological importance. It also fails to consider valuable information such as the biological properties of genes, the relationships between gene regulatory networks and the functions and significance of genes. To address these limitations, we conducted a more detailed analysis of YARS1 using GSEA. According to our research, it has been discovered that the immune microenvironment of bladder cancer is significantly associated with YARS1, specifically the signalling pathways of the B cell receptor and T cell receptor (Figure 4A, B). Furthermore, YARS1 was involved in the intestinal immune network for IgA production and the CD8 Tcr pathway (Figure 4C, D). In bladder cancer, YARS1 plays a critical role in regulating MYC, TP53, PLK1 and PD1 (Figure 4E–H). Furthermore, through GSEA, we confirmed significant associations between YARS1 and DNA methylation, ferroptosis, stem pathway and senescence (Figure 4I–L). Finally, we analysed YARS1 interacting proteins that were common to these four genes, as well as the target genes, using the Gendoma database (Figure 4M–P). In conclusion, our detailed GSEA provides insights into the multiple potential functions of YARS1 in bladder cancer.

### 3.5 Constructing an immune cell‐related prognostic model based on LASSO

**FIGURE 5:**
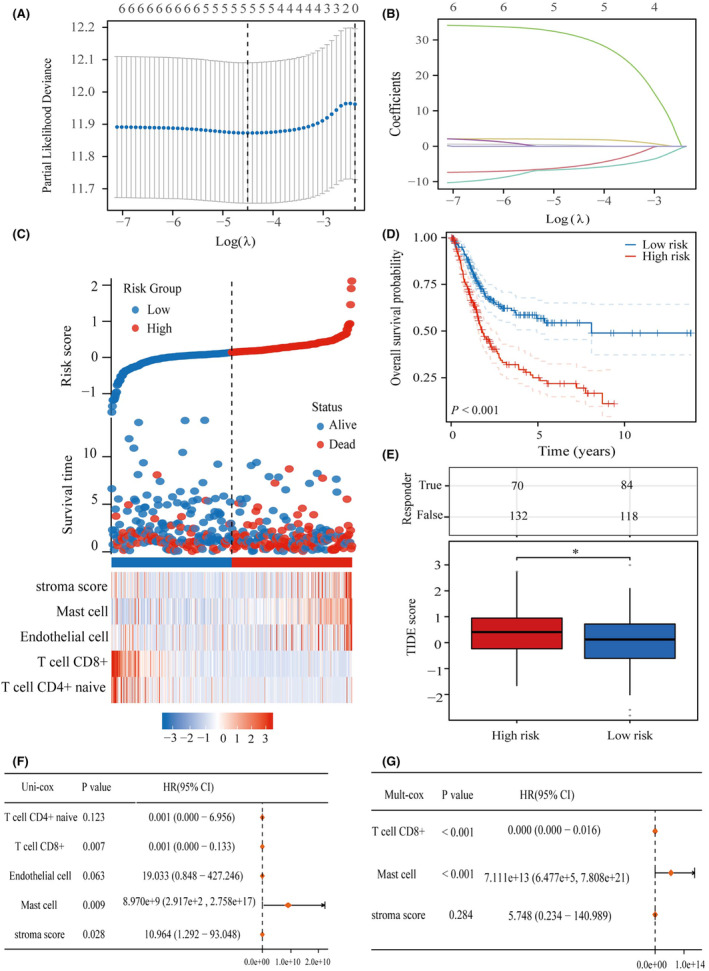
Elaboration of a predictive model for immune cells. (A) Identification of seven immune cells utilizing LASSO technique. (B) Incorporation of five immune cells into the predictive model. (C) Demonstration of five predictive cells linked to immune infiltration along with their expression patterns in patients with BLCA having diverse risk scores and distinct survival outcomes. (D) KM curves showcasing the division of participants into high‐ and low‐risk groups. (E) Assessment of the responsiveness of Immune Checkpoint Inhibitors in high‐ and low‐risk groups. (F) Examination of the prognostic significance of five distinct immune cell types through univariate cox regression analysis. (G) Evaluation of the prognostic significance of five distinct immune cell types through multifactorial cox regression analysis. *p < .05.

The TCGA database bladder cancer data set was analysed using the XCELL algorithm, which assigned scores to 38 immune cells. Of these, only seven immune cell types were significantly associated with bladder cancer prognosis (Figure S1). Our prognostic model incorporated five immune cell types (Figure 5A, B): CD4^+^ naïve T cells CD8^+^, endothelial cells, mast cells and stroma. To calculate the risk score for the model, the following equation was used: Risk score = (−5.548) * T cell CD4^+^ naive + (−6.347) * T cell CD8^+^ + (0.287) * Endothelial cell + (30.935) * Mast cell + (1.922) * stroma score. By examining the expression patterns of these five genes across different risk scores, survival rates and patient outcomes (Figure 5C), we observed that patients in the high‐risk group had a significantly worse prognosis than those in the low‐risk group (Figure 5D). Furthermore, we evaluated the responsiveness of our prognostic model to immune checkpoint inhibitors using the Tumor Immune Dysfunction and Exclusion (TIDE) algorithm. Interestingly, the patients in the high‐risk group exhibited significantly higher TIDE scores than those in the low‐risk group, suggesting a reduced response to immune checkpoint inhibitors (Figure 5E). Additional investigations involved univariate multifactorial cox regression analysis to examine the prognostic characteristics of the five immune cells in the model. The results of this analysis confirmed that CD8^+^ T cells and mast cells are potential prognostic biomarkers for bladder cancer (Figure 5F, G). These findings substantiate the prognostic importance of immune cells in patients with bladder cancer.

### 3.6 Analysis of prognostic models correlating with bladder cancer immunotherapy

**FIGURE 6:**
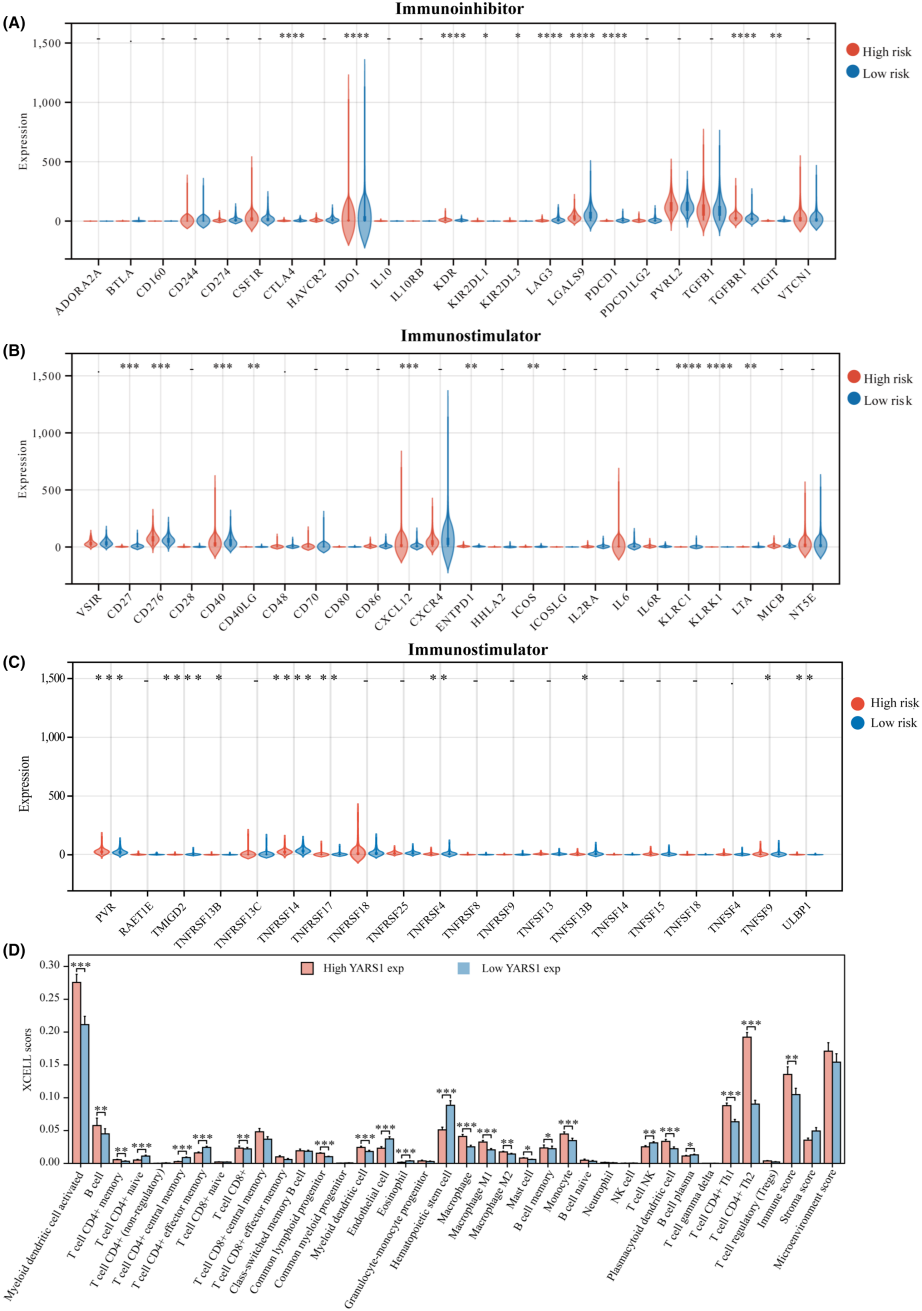
Correlation between prognostic models and immunostimulator and immunoinhibitor. (A–C) Correlation between prognostic models and immunostimulator and immunoinhibitory. (D) Correlation between YARS1 expression and immune cells. *p < .05, **p < .01, ***p < .001, ****p < .0001.

Our study involved an extensive analysis of the expression of factors that suppress or stimulate the immune system in two distinct groups: high and low risk. We discovered noteworthy variations in the expression of certain genes, namely CTLA4, IDO1, KDR, KIR2DL1, KIR2DL3, LAG3, LGALS9, PDCD1, TGFBR1 and TIGIT, between the two groups (Figure 6A). Additionally, we observed significant differences in the expression of CD27, CD276, CD40, CD40LG, CXCL12, ENTPD1, ICOS, KLRC1, KLRK1, PVR, TMIGD2, TNFRSF13B, TNFRSF14, TNFRSF17, TNFRSF4, TNFSF13B, TNFSF9 and ULBP1 between the high‐ and low‐risk groups (Figure 6B, C). Finally, in addition to our investigation of YARS1, we explored its correlation with immune cells in bladder cancer. The analysis revealed considerable differences in the scores of the 24 immune cells between the YARS1 high‐expression and the YARS1 low‐expression groups (Figure 6D). Our results confirm the important role of YARS1 in the immune microenvironment of bladder cancer, as YARS1 is not only significantly associated with a variety of immunostimulatory and immunoinhibitor factors, but also there is a significant relationship between YARS1 and a variety of immune cell infiltrations.

### 3.7 Analysis of the correlation between PRPF19 and immune cell infiltration in bladder cancer

**FIGURE 7:**
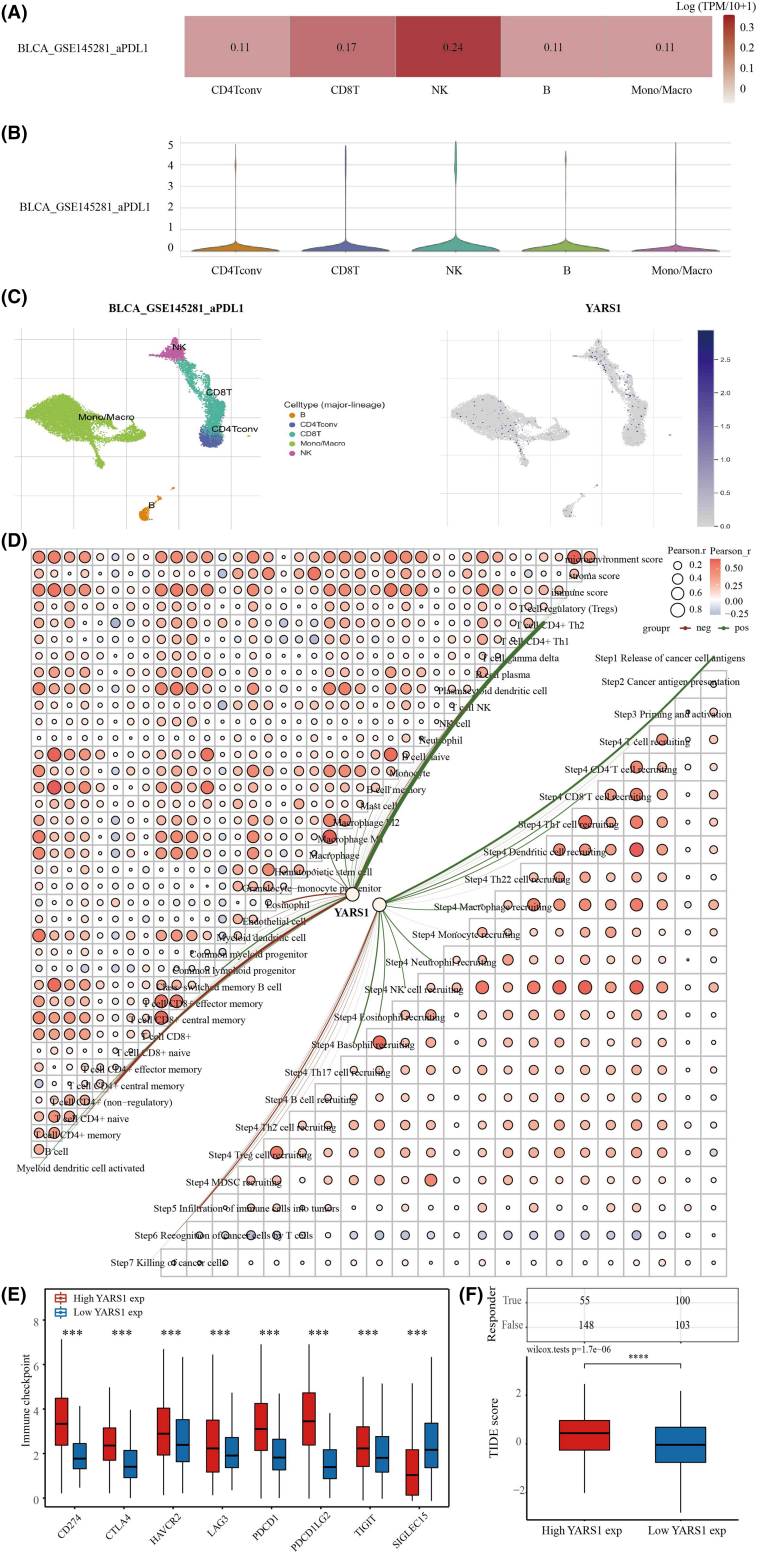
YARS1 is closely related to the immune microenvironment of bladder cancer. (A–C) Analysis of YARS1 and bladder cancer immune cell infiltration levels in the GSE145281 data set. (D) Correlation between YARS1 expression and immunity scores and correlation between immunity scores themselves Network plot. (E) Correlation analysis of YARS1 expression with immune checkpoint genes. (F) Analysis of YARS1 expression and response to immune checkpoint inhibitor therapy. ***p < .001, ****p < .0001.

First, the correlation between YARS1 and immune cell infiltration in bladder cancer was explored using single‐cell analysis. In the GSE145281 data set, YARS1 significantly correlated with CD4Tconv, CD8T cells, NK cells, B cells and mono/macro cells (Figure 7A, B). Heat maps were used to illustrate the correlation between YARS1 and different immune cells (Figure 7C). Furthermore, the correlation between YARS1 expression and immunity scores based on the XCELL and TIP algorithms was demonstrated, along with the correlation analysis between the immunity scores, using correlation network graphs (Figure 7D). Previous research has demonstrated the prognostic value of CD8^+^ T cells, and this study identified a regulatory effect of YARS1 on CD8^+^ T cells, suggesting that YARS1 may affect the prognosis of patients with bladder cancer by influencing CD8^+^ T cells. Furthermore, we examined the correlation between YARS1 and immune checkpoint genes and revealed significant differences in all immune checkpoint genes between the YARS1 high‐ and low‐expression groups (Figure 7E). Finally, we investigated the correlation between YARS1 expression and immune checkpoint inhibitor treatment using the TIDE algorithm. The TIDE scores of patients in the YARS1 high‐expression group were significantly higher than those in the YARS1 low‐expression group, indicating that patients with high YARS1 expression had poorer outcomes with immune checkpoint inhibitor treatment (Figure 7F). In summary, this study establishes a robust association between YARS1 expression and immunotherapy in patients with bladder cancer.

### 3.8 Analysis of YARS1 methylation levels

**FIGURE 8:**
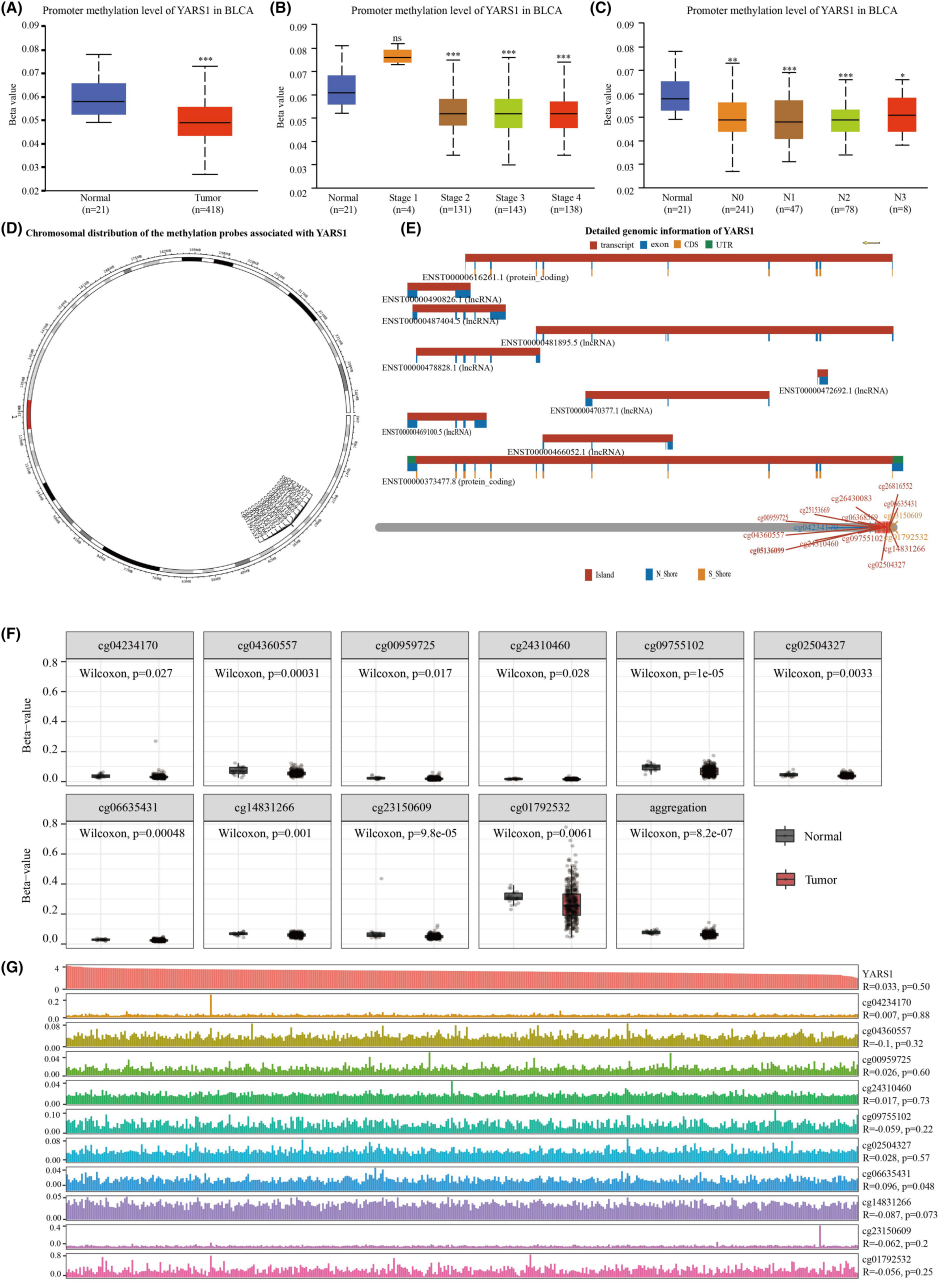
The methylation levels of YARS1 DNA exhibit a significant decrease in bladder cancer compared to normal bladder samples. (A) A comparison was made between the DNA methylation levels of YARS1 in bladder cancer and normal bladder samples. (B) The DNA methylation levels of YARS1 were compared across different stages of bladder cancer. (C) The DNA methylation levels of YARS1 were compared across different N‐stages of bladder cancer. (D) The distribution of methylation probes related to YARS1 on the chromosomal level was analysed. (E) Detailed genomic information regarding YARS1 was provided. (F) The differential expression of methylation probes associated with YARS1 was examined between bladder cancer and normal bladder samples. (G) A correlation analysis was conducted between YARS1‐associated methylation probes and YARS1 expression in bladder cancer. ns = *p* < .05, *p < .05, **p < .01, ***p < .001.

Gene enrichment analysis based on YARS1 revealed a strong association between YARS1 and DNA methylation in bladder cancer. To delve deeper into this relationship, we conducted a thorough investigation of variations in YARS1 DNA methylation levels in bladder cancer. Our findings revealed that the DNA methylation levels of YARS1 were significantly lower in bladder cancer samples than in normal bladder samples (Figure 8A). Notably, the methylation levels of YARS1 underwent noteworthy changes upon reaching pathological stage 2. Furthermore, we compared the methylation levels of patients with the N stage to those of normal samples (Figure 8B, C). To gain deeper insight into the role of YARS1 methylation in bladder cancer, we used the SMART database. Initially, we presented the distribution of methylation probes associated with YARS1 on chromosomes in bladder cancer, followed by an analysis of the extensive genomic information linked to YARS1 (Figure 8D, E). Our analysis unearthed a total of 10 methylation probes related to YARS1 that displayed substantial differences between bladder cancer and normal bladder samples (Figure 8F). Finally, we explored the correlation between these methylation probes and YARS1 expression in bladder cancer. Interestingly, we observed a negative correlation between cg04360557 and YARS1, whereas cg06635431 was positively correlated with YARS1 (Figure 8F). In conclusion, we analysed the correlation between YARS1 and different methylation probes in bladder cancer, and suggest that YARS1 may play an oncogenic role in bladder cancer through these methylation probes.

### 3.9 Correlation analysis of YARS1 with ferroptosis, senescence and stemness characteristics in bladder cancer

**FIGURE 9:**
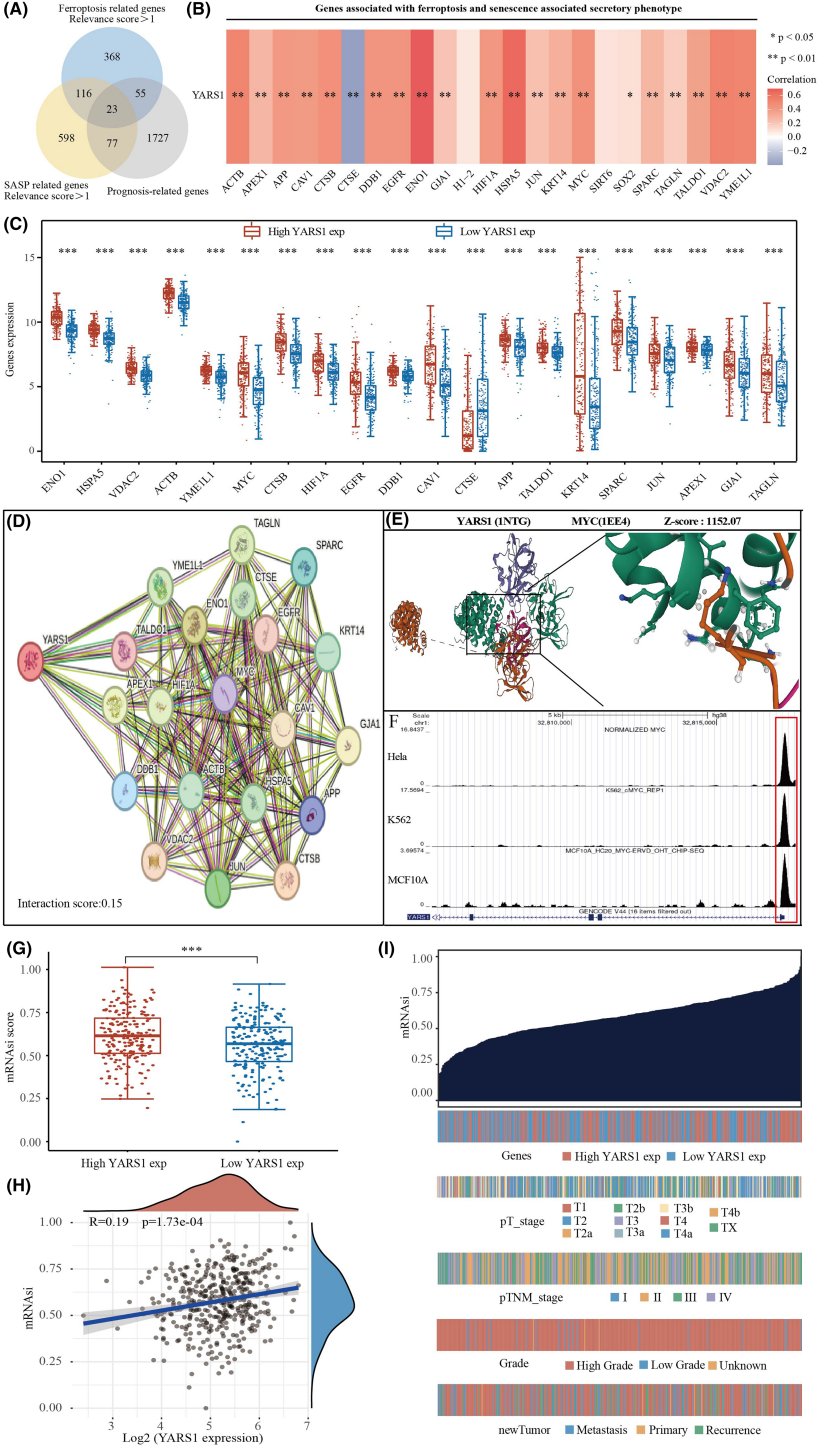
YARS1 interacts with MYC to regulate ferroptosis, senescence and stemness in bladder cancer cells. (A) Identification of ferroptosis and cellular senescence‐related prognostic genes. (B) Analysis of ferroptosis and cellular senescence‐related prognostic genes correlating with YARS1. (C) Differential analysis of ferroptosis and cell senescence‐related prognostic genes in high YARS1 expression group and low YARS1 expression group. (D) Interaction network of YARS1. (E) Molecular docking of YARS1 with MYC. (F) MYC regulates the promoter activity of YARS1. (G, H) YARS1 positively correlates with bladder cancer stemness score. (I) Heat map of bladder cancer stemness score. *p < .05, **p < .01, ***p < .001.

Gene enrichment analyses revealed that YARS1 may play a role in regulating the senescence, ferroptosis and stemness pathways in bladder cancer cells. Previous studies have demonstrated the regulation of stemness through ferroptosis and cellular senescence. Therefore, we aimed to identify key genes that could potentially target both ferroptosis and cellular senescence in the context of bladder cancer cell stemness. To achieve this, we used the Genecards website to obtain a list of ferroptosis‐ and cellular senescence‐related genes. From this list, we selected genes with correlation coefficients greater than one for further investigation. Ultimately, 23 key prognostic genes were identified (Figure 9A). Among these genes, YARS1 showed no significant correlation with H1‐2 or SIRT6, a negative correlation with CTSE and a positive correlation with the other 20 genes (Figure 9B). Subsequently, we analysed the differences in the expression of these 23 genes between the YARS1 high‐ and low‐expression groups. We observed significant differences in the expression of all 20 genes, except H1‐2, SIRT6 and SOX2 (Figure 9C). Furthermore, we identified 10 genes that could interact with YARS1 among these differentially expressed genes (Figure 9D). Combined with the results of the gene enrichment analysis of YARS1, we investigated the potential oncogenic role of YARS1 through its interaction with MYC. To confirm this interaction, we conducted a molecular docking analysis, which revealed perfect docking between YARS1 and MYC in the molecular structure (Figure 9E). Because MYC is both a well‐known oncogene and a crucial transcription factor, we further demonstrated that MYC regulates the promoter region of YARS1 (Figure 9F). Considering that ferroptosis and cellular senescence can affect the stemness characteristics of tumour cells, we also examined the effect of YARS1 on bladder cancer stemness. Our findings indicated a positive correlation between high YARS1 expression and increased stemness scores in bladder cancer samples (Figure 9G, H). Furthermore, we visualized the dryness score of the bladder cancer samples using a heat map in conjunction with the clinical stage, grading and other factors of bladder cancer (Figure 9I). In conclusion, our study suggests that YARS1 modulates ferroptosis, senescence and stemness in bladder cancer cells by interacting with MYC.

### 3.10 Building a YARS1‐associated ceRNA network

**FIGURE 10:**
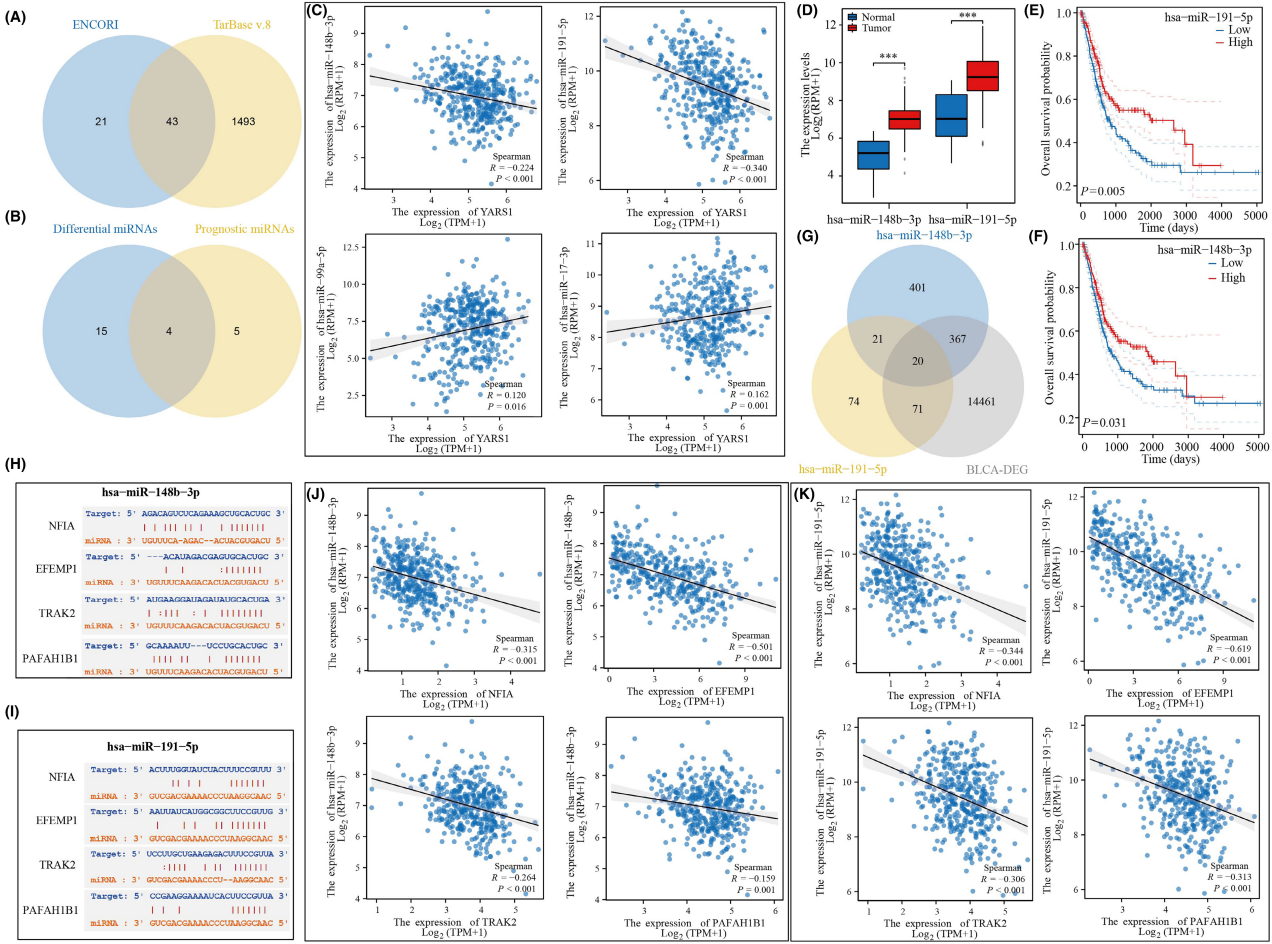
Construction of a bladder cancer prognosis‐related ceRNA network. (A) ENCORI and TarBase databases predict YARS1‐related miRNAs. (B) Intersection of YARS1‐related miRNAs of difference and prognostic miRNAs in bladder cancer. (C) Correlation of YARS1‐related miRNAs for differential prognosis with YARS1 in bladder cancer. (D) Expression of YARS1‐related miRNAs in bladder cancer. (E, F) Prognostic value of YARS1‐related miRNAs in bladder cancer. (G) Targeted circRNA screening for YARS1‐related miRNAs. (H, I) Gene sequences of miRNA‐related circRNAs. (J, K) Analysis of miRNA‐associated circRNA correlation with miRNAs. ***p＜0.001.

We identified 64 miRNAs with targeting relationships for YARS1 using the ENCORI database and 1536 miRNAs using the TarBase database. From these data sets, we selected 43 miRNAs that were common to both data sets (Figure 10A). Of these 43 miRNAs, four were found to be bladder cancer prognostic differential miRNAs (Figure 10B). We analysed the correlation between these four miRNAs and YARS1 expression in bladder cancer. As expected, miRNAs were negatively correlated with their target genes, and hsa‐miR‐148b‐3p and hsa‐miR‐191‐5p were negatively correlated with YARS1 in bladder cancer (Figure 10C). The expression levels of hsa‐miR‐148b‐3p and hsa‐miR‐191‐5p were significantly higher in bladder cancer samples than in normal bladder samples, and low expression of both miRNAs was associated with poor patient prognosis (Figure 10D–F). Additionally, we analysed circRNAs that could target these two miRNAs and identified 20 circRNAs that showed differential expression in bladder cancer samples compared with normal bladder samples (Figure 10G). Of these 20 circRNAs, only four were associated with bladder cancer prognosis (Figure S2). We also provided sequence information for hsa‐miR‐148b‐3p and hsa‐miR‐191‐5p along with these four circRNAs (Figure 10H, I). Finally, we examined the correlation between these four circRNAs and hsa‐miR‐148b‐3p/hsa‐miR‐191‐5p in bladder cancer and found that they were negatively correlated (Figure 10J, K). In conclusion, our analysis confirmed that the ceRNA network YARS1‐hsa‐miR‐148b‐3p/hsa‐miR‐191‐5p‐NFIA/EFEMP1/TRAK2/PAFAH1B1 is associated with bladder cancer prognosis.

## 4 DISCUSSION

YARS, a gene involved in maintaining vital biological activities, plays a notable role in facilitating tyrosylation.^17^ However, the role of YARS1 in cancer remains unclear. Previous studies on gastric cancer and hepatocellular carcinoma have provided evidence of YARS1's pro‐cancer effects. In the diagnosis of hepatocellular carcinoma, YARS1 exhibits clinical significance and enhances cancer progression by activating the PI3K/AKT signalling pathway.^18^ The objective of this study was to explore the expression and prognostic relevance of YARS1 in bladder cancer using diverse bioinformatics methodologies and to analyse its biological functions.

The expression of YARS1 in various grades of bladder cancer was examined using the TCGA‐BLCA data set. Furthermore, we cross‐validated our findings using the GSE13507 data set. Our results demonstrated that YARS1 exhibited a substantial increase in expression in bladder cancer, particularly in high‐grade tumours, thereby implying its potential as an oncogene in bladder cancer. Furthermore, an assessment was conducted to ascertain the prognostic significance of YARS1 in bladder cancer, and the results demonstrated that patients with heightened YARS1 expression had an unfavourable prognosis. This observation remained consistent in both TCGA‐BLCA and GSE13507 data sets, where individuals with elevated YARS1 expression had lower overall survival rates and lower disease‐specific survival rates. Further validation was conducted using multifactorial COX regression analyses, which confirmed the prognostic relevance of YARS1 in both data sets. Based on these findings, we conclude that YARS1 holds promise as a valuable prognostic biomarker for patients diagnosed with bladder cancer.

To explore the biological role of YARS1, we performed gene enrichment analysis on samples from patients with bladder cancer. Both KEGG and GSEA findings confirmed a robust connection between YARS1 and the immune microenvironment of bladder cancer. Components of the tumour microenvironment include immune cells, cytokines and chemokines that promote immune escape, tumour growth and metastasis. Studying these components and their complex interactions will provide insights into their potential oncogenic mechanisms.^19^ Therefore, we conducted subsequent analyses of immune infiltration. Using the xCell algorithm, we gained insight into the infiltration of 38 immune cells known for their crucial involvement in the progression and treatment of bladder cancer. Among these cells, seven were identified as prognostic markers for patients with bladder cancer, of which five were incorporated into the prognostic model. Immunotherapy has shown potential efficacy in a wide range of tumours, introducing new therapeutic alternatives for otherwise incurable patients.^20^ Cancer immunotherapy, which eliminates tumour cells by modulating the patient's immune system, has revolutionized cancer treatment.^21^ Therefore, assessing the responsiveness of patients with bladder cancer to immunotherapy is of great clinical value. Notably, the high‐risk group had a significantly poorer prognosis than the low‐risk group. We used the TIDE algorithm to evaluate the responsiveness of the two groups to immune checkpoint inhibitor therapy. Interestingly, patients in the high‐risk group exhibited inadequate response to immune checkpoint inhibitors, potentially explaining their unfavourable prognoses. Moreover, multifactorial COX regression analysis revealed that infiltration of CD8^+^ T cells and mast cells played a role as prognostic biomarkers of bladder cancer. Recent investigations have indicated that reducing the infiltration and activity of CD8^+^ T cells can promote resistance to immunotherapy in bladder cancer,^22^ whereas mast cell density has been shown to predict lymph node metastasis in patients with breast cancer.^23^ Interestingly, we observed a correlation between YARS1 expression and the degree of CD8^+^ T‐cell and mast cell infiltration. Additionally, patients with high YARS1 expression displayed a poor response to immune checkpoint inhibitor therapy. Therefore, it is plausible that the regulation of YARS1 in bladder cancer immunotherapy may also be associated with the behaviour of these two immune cells.

Aberrant DNA methylation is regarded as a promising biomarker with potential applications in the diagnosis, prognosis and treatment of bladder cancer.^24^, ^25^ DNA methylation, one of the earliest discovered mechanisms of epigenetic gene modification, plays a critical role in maintaining normal cellular function, preserving chromosomal structure, facilitating X‐chromosome inactivation, regulating gene imprinting, controlling embryonic developmental processes, influencing ageing and contributing to cancer development.^26^ Tumours often exhibit altered DNA methylation patterns characterized by a reduction in overall methylation levels and an increase in local methylation levels. Hypomethylated states activate oncogenes.^27^ Our findings suggest that the methylation levels of YARS1 are significantly lower in bladder cancer samples than in healthy bladder samples. Therefore, we propose that decreased methylation of YARS1 may be linked to its role in promoting bladder cancer progression.

Ferroptosis, an iron‐dependent programmed cell death, is a multifaceted process.^28^, ^29^ Recent investigations have shed light on the impact of the ferroptotic pathway on the prognosis and immunotherapy responses of individuals diagnosed with bladder cancer.^30^ Additionally, the induction of ferroptosis has proven to be effective in overcoming the chemotherapy resistance exhibited by bladder cancer cells.^31^ Moreover, induction of ferroptosis in various tumours inhibits tumour stemness.^32^, ^33^, ^34^, ^35^ It has been discovered that many of the same molecular signalling networks regulate both cellular senescence and the maintenance of stem cell stemness. Key effector signalling molecules involved in the regulation of cellular senescence, such as Bmi‐1, p16, p21, p53 and H3K9me3, are also involved in maintaining stem cell stemness.^36^ In conclusion, ferroptosis and cellular senescence are closely linked to the maintenance of tumour cell stemness. Therefore, we integrated genes that regulate both ferroptosis and cellular senescence and analysed their correlation with YARS1 expression. Interestingly, our analysis revealed a significant correlation between YARS1 and 21 genes that co‐regulate ferroptosis and cellular senescence in bladder cancer samples. Among these genes, MYC was found to interact with YARS1 in bladder cancer and to regulate cellular senescence and ferroptosis. MYC, a well‐known oncogene, has been extensively studied in tumour research. Recent studies have also reported its important role in response to tumour stemness and ferroptosis regulation.^37^ Furthermore, our previous study has confirmed that MYC regulates senescence in bladder cancer cells.^38^ Using molecular docking methods, we demonstrated the interaction between YARS1 and MYC, as well as the regulation of YARS1 promoter activity by MYC. Based on these findings, we hypothesized that YARS1 interacts with MYC to regulate bladder cancer cell senescence, ferroptosis and stemness. However, further confirmation is required using bioinformatics methods as well as cellular and animal experiments. Finally, we constructed a ceRNA network based on YARS1. The ceRNA network is a form of post‐transcriptional regulation mediated by miRNAs that links the functionalities of coding and non‐coding RNA molecules. Through competitive binding of lncRNAs or circular RNAs with miRNAs, the network manages the expression of mRNA, thus affecting various biological processes and leading to the emergence of diverse diseases.^39^ In this study, we successfully identified two miRNAs that interact with YARS1 using differential expression and survival analysis techniques. Our screening efforts revealed four additional circRNAs that specifically targeted hsa‐miR‐148b‐3p and hsa‐miR‐191‐5p. In summary, the established ceRNA network exhibited a substantial correlation with the prognosis of bladder cancer.

## 5 CONCLUSION

The differential expression of YARS1 in bladder cancer is substantiated in this study. These findings establish YARS1 as a promising prognostic biomarker of this disease. Furthermore, our investigations revealed an intricate interaction between YARS1 and MYC in the regulation of bladder cancer cell senescence, ferroptosis and stemness. Ultimately, the identification of a specific ceRNA network paves the way for potential therapeutic interventions for bladder cancer.

## AUTHOR CONTRIBUTIONS

**Yaxuan Wang:** Conceptualization (lead); validation (lead); writing – original draft (lead). **Jinfeng Wang:** Conceptualization (equal); data curation (equal); software (equal). **Lu Zhang:** Investigation (equal); methodology (equal); visualization (equal). **JiaXing He:** Conceptualization (supporting); resources (supporting). **Bo Ji:** Data curation (equal); investigation (equal). **JianShe Wang:** Data curation (equal); investigation (equal). **BeiChen Ding:** Supervision (equal); writing – review and editing (equal). **MingHua Ren:** Funding acquisition (equal); writing – review and editing (equal).

## CONFLICT OF INTEREST STATEMENT

The authors declare that the research was conducted without any commercial or financial relationships that could be construed as a potential conflict of interest.

## Supporting information


Figure S1.

Figure S2.

## Data Availability

The data sets obtained from TCGA database (https://portal.gdc.cancer.gov/) and UALCAN database (http://ualcan.path.uab.edu/analysis.html), GEO database (https://www.ncbi.nlm.nih.gov/geo/), partial analysis by Cbioportal for Cancer Genomics website (http://www.cbioportal.org), TISCH (http://tisch.comp‐genomics.org/home/) database and SMART database (http://www.bioinfo‐zs.com/smartapp/).

## References

[1] Liu T , Fan MQ , Xie XX , et al. Activation of CTNNB1 by deubiquitinase UCHL3‐mediated stabilization facilitates bladder cancer progression. J Transl Med. 2023;21(1):656. doi:10.1186/s12967-023-04311-3 37740194 PMC10517567

[2] Jiang LJ , Guo SB , Huang ZY , et al. PHB promotes bladder cancer cell epithelial‐mesenchymal transition via the Wnt/β‐catenin signaling pathway. Pathol Res Pract. 2023;247:154536. doi:10.1016/j.prp.2023.154536 37235908

[3] Siracusano S , Rizzetto R , Porcaro AB . Bladder cancer genomics. Urologia. 2020;87(2):49‐56. doi:10.1177/0391560319899011 31942831

[4] Carter CW Jr . Cognition, mechanism, and evolutionary relationships in aminoacyl‐tRNA synthetases. Annu Rev Biochem. 1993;62:715‐748. doi:10.1146/annurev.bi.62.070193.003435 8352600

[5] Sun J , Lv PC , Zhu HL . Tyrosyl‐tRNA synthetase inhibitors: a patent review. Expert Opin Ther Pat. 2017;27(5):557‐564. doi:10.1080/13543776.2017.1273350 27977303

[6] Hyeon DY , Kim JH , Ahn TJ , Cho Y , Hwang D , Kim S . Evolution of the multi‐tRNA synthetase complex and its role in cancer. J Biol Chem. 2019;294(14):5340‐5351. doi:10.1074/jbc.REV118.002958 30782841 PMC6462501

[7] Jordanova A , Irobi J , Thomas FP , et al. Disrupted function and axonal distribution of mutant tyrosyl‐tRNA synthetase in dominant intermediate Charcot‐Marie‐tooth neuropathy. Nat Genet. 2006;38(2):197‐202. doi:10.1038/ng1727 16429158

[8] Zhang C , Lin X , Zhao Q , et al. YARS as an oncogenic protein that promotes gastric cancer progression through activating PI3K‐Akt signaling. J Cancer Res Clin Oncol. 2020;146(2):329‐342. doi:10.1007/s00432-019-03115-7 31912229 PMC6985085

[9] Hao H , Wang Z , Ren S , et al. Reduced GRAMD1C expression correlates to poor prognosis and immune infiltrates in kidney renal clear cell carcinoma. PeerJ. 2019;7:e8205. doi:10.7717/peerj.8205 31875150 PMC6927341

[10] Sturm G , Finotello F , Petitprez F , et al. Comprehensive evaluation of transcriptome‐based cell‐type quantification methods for immuno‐oncology. Bioinformatics. 2019;35(14):i436‐i445. doi:10.1093/bioinformatics/btz363 31510660 PMC6612828

[11] Newman AM , Liu CL , Green MR , et al. Robust enumeration of cell subsets from tissue expression profiles. Nat Methods. 2015;12(5):453‐457. doi:10.1038/nmeth.3337 25822800 PMC4739640

[12] Chandrashekar DS , Karthikeyan SK , Korla PK , et al. UALCAN: an update to the integrated cancer data analysis platform. Neoplasia. 2022;25:18‐27. doi:10.1016/j.neo.2022.01.001 35078134 PMC8788199

[13] Li Y , Ge D , Lu C . The SMART app: an interactive web application for comprehensive DNA methylation analysis and visualization. Epigenetics Chromatin. 2019;12(1):71. doi:10.1186/s13072-019-0316-3 31805986 PMC6894252

[14] Malta TM , Sokolov A , Gentles AJ , et al. Machine learning identifies stemness features associated with oncogenic dedifferentiation. Cell. 2018;173(2):338‐354.e15. doi:10.1016/j.cell.2018.03.034 29625051 PMC5902191

[15] Li JH , Liu S , Zhou H , Qu LH , Yang JH . starBase v2.0: decoding miRNA‐ceRNA, miRNA‐ncRNA and protein‐RNA interaction networks from large‐scale CLIP‐seq data. Nucleic Acids Res. 2014;42(Database issue):D92‐D97. doi:10.1093/nar/gkt1248 24297251 PMC3964941

[16] Karagkouni D , Paraskevopoulou MD , Chatzopoulos S , et al. DIANA‐TarBase v8: a decade‐long collection of experimentally supported miRNA‐gene interactions. Nucleic Acids Res. 2018;46(D1):D239‐D245. doi:10.1093/nar/gkx1141 29156006 PMC5753203

[17] Gonzaga‐Jauregui C , Harel T , Gambin T , et al. Exome sequence analysis suggests that genetic burden contributes to phenotypic variability and complex neuropathy. Cell Rep. 2015;12(7):1169‐1183. doi:10.1016/j.celrep.2015.07.023 26257172 PMC4545408

[18] Zhou W , Wang J , Zhang J , et al. LncRNA NCK1‐AS1 aggravates hepatocellular carcinoma by the miR‐22‐3p/YARS Axis to activate PI3K/AKT signaling. J Gastrointestin Liver Dis. 2022;31(1):48‐59. doi:10.15403/jgld-4077 35306563

[19] Ren S , Wang W , Shen H , et al. Development and validation of a clinical prognostic model based on immune‐related genes expressed in clear cell renal cell carcinoma. Front Oncol. 2020;10:1496. doi:10.3389/fonc.2020.01496 32983989 PMC7485294

[20] Guo SB , Pan DQ , Su N , et al. Comprehensive scientometrics and visualization study profiles lymphoma metabolism and identifies its significant research signatures. Front Endocrinol (Lausanne). 2023;14:1266721. doi:10.3389/fendo.2023.1266721 37822596 PMC10562636

[21] Guo SB , Du S , Cai KY , Cai HJ , Huang WJ , Tian XP . A scientometrics and visualization analysis of oxidative stress modulator Nrf2 in cancer profiles its characteristics and reveals its association with immune response. Heliyon. 2023;9(6):e17075. doi:10.1016/j.heliyon.2023.e17075 37342570 PMC10277599

[22] Yu A , Hu J , Fu L , et al. Bladder cancer intrinsic LRFN2 drives anticancer immunotherapy resistance by attenuating CD8^+^ T cell infiltration and functional transition. J Immunother Cancer. 2023;11(10):e007230. doi:10.1136/jitc-2023-007230 37802603 PMC10565151

[23] Floroni E , Ceauşu AR , Cosoroabă RM , et al. Mast cell density in the primary tumor predicts lymph node metastases in patients with breast cancer. Rom J Morphol Embryol. 2022;63(1):129‐135. doi:10.47162/RJME.63.1.13 36074676 PMC9593109

[24] Larsen LK , Lind GE , Guldberg P , Dahl C . DNA‐methylation‐based detection of urological cancer in urine: overview of biomarkers and considerations on biomarker design, source of DNA, and detection technologies. Int J Mol Sci. 2019;20(11):2657. doi:10.3390/ijms20112657 31151158 PMC6600406

[25] Padrão NA , Monteiro‐Reis S , Torres‐Ferreira J , et al. MicroRNA promoter methylation: a new tool for accurate detection of urothelial carcinoma. Br J Cancer. 2017;116(5):634‐639. doi:10.1038/bjc.2016.454 28081549 PMC5344289

[26] Liu J , Huang B , Ding F , Li Y . Environment factors, DNA methylation, and cancer. Environ Geochem Health. 2023;45(11):7543‐7568. doi:10.1007/s10653-023-01749-8 37715840

[27] Yuan T , Edelmann D , Fan Z , et al. Machine learning in the identification of prognostic DNA methylation biomarkers among patients with cancer: a systematic review of epigenome‐wide studies. Artif Intell Med. 2023;143:102589. doi:10.1016/j.artmed.2023.102589 37673571

[28] Dixon SJ , Lemberg KM , Lamprecht MR , et al. Ferroptosis: an iron‐dependent form of nonapoptotic cell death. Cell. 2012;149(5):1060‐1072. doi:10.1016/j.cell.2012.03.042 22632970 PMC3367386

[29] Shan Z , Tang W , Shi Z , Shan T . Ferroptosis: An emerging target for bladder cancer therapy. Curr Issues Mol Biol. 2023;45(10):8201‐8214. doi:10.3390/cimb45100517 37886960 PMC10605744

[30] Liu X , Qiu Z , Zhang X , et al. Generalized machine learning based on multi‐omics data to profile the effect of ferroptosis pathway on prognosis and immunotherapy response in patients with bladder cancer. Environ Toxicol. 2023;39:680‐694. doi:10.1002/tox.23949 37647346

[31] Zhao Y , Ren P , Yang Z , Wang L , Hu C . Inhibition of SND1 overcomes chemoresistance in bladder cancer cells by promoting ferroptosis. Oncol Rep. 2023;49(1):16. doi:10.3892/or.2022.8453 36453257 PMC9773013

[32] Lv J , Wang Z , Liu H . Erianin suppressed lung cancer stemness and chemotherapeutic sensitivity via triggering ferroptosis. Environ Toxicol. 2023;39:479‐486. doi:10.1002/tox.23832 37209271

[33] Yang Y , Lu Y , Zhang C , et al. Phenazine derivatives attenuate the stemness of breast cancer cells through triggering ferroptosis. Cell Mol Life Sci. 2022;79(7):360. doi:10.1007/s00018-022-04384-1 35690642 PMC11072418

[34] Zhao X , Zhou M , Yang Y , Luo M . The ubiquitin hydrolase OTUB1 promotes glioma cell stemness via suppressing ferroptosis through stabilizing SLC7A11 protein. Bioengineered. 2021;12(2):12636‐12645. doi:10.1080/21655979.2021.2011633 34927544 PMC8810032

[35] Zhang H , Wang M , He Y , et al. Chemotoxicity‐induced exosomal lncFERO regulates ferroptosis and stemness in gastric cancer stem cells. Cell Death Dis. 2021;12(12):1116. doi:10.1038/s41419-021-04406-z 34845198 PMC8629982

[36] Milanovic M , Fan DNY , Belenki D , et al. Senescence‐associated reprogramming promotes cancer stemness. Nature. 2018;553(7686):96‐100. doi:10.1038/nature25167 29258294

[37] Wang Z , Ouyang L , Liu N , et al. The DUBA‐SLC7A11‐c‐Myc axis is critical for stemness and ferroptosis. Oncogene. 2023;42(36):2688‐2700. doi:10.1038/s41388-023-02744-0 37537342

[38] Wang Y , Zhu H , Xu H , Qiu Y , Zhu Y , Wang X . Senescence‐related gene c‐Myc affects bladder cancer cell senescence by interacting with HSP90B1 to regulate cisplatin sensitivity. Aging (Albany NY). 2023;15(15):7408‐7423. doi:10.18632/aging.204863 37433010 PMC10457043

[39] Lu J , Kang X , Wang Z , Zhao G , Jiang B . The activity level of follicular helper T cells in the peripheral blood of osteosarcoma patients is associated with poor prognosis. Bioengineered. 2022;13(2):3751‐3759. doi:10.1080/21655979.2022.2031387 35081874 PMC8974108

