# Supplementary material for: Unveiling the role of YARS1 in bladder cancer: A prognostic biomarker and therapeutic target
Source: J Cell Mol Med. 2024 Mar 20;28(7):e18213. doi: 10.1111/jcmm.18213 (PMC10951887; doi:10.1111/jcmm.18213)
Supplement: Supplementary file 1 — Figure S1. [file JCMM-28-e18213-s002.docx]

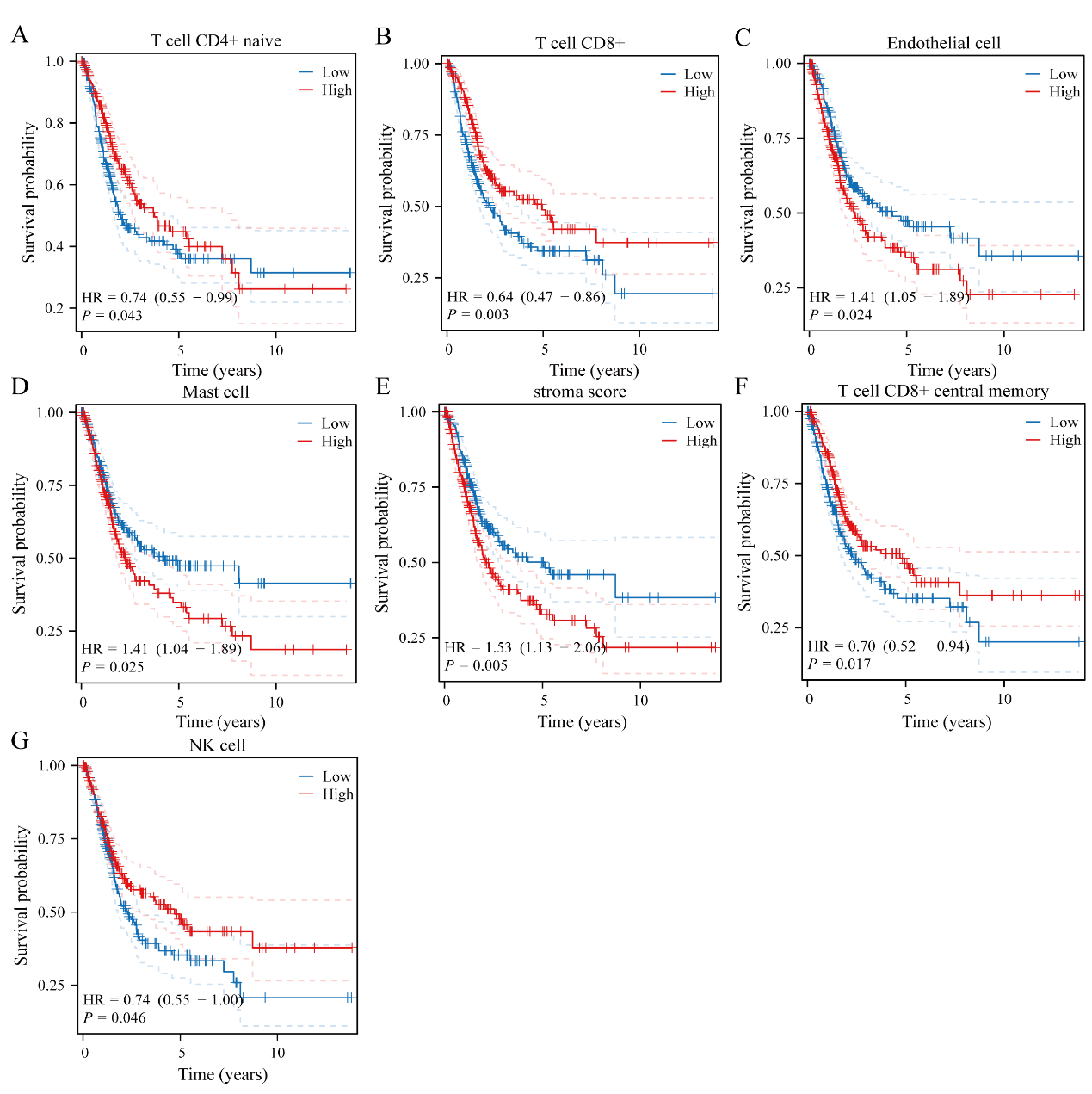


Supplementary figure 1 Analysis of immune infiltration level scores based on the XCELL algorithm for their prognostic relevance to BLCA. (A-G) Relationship between different immune cell infiltration level scores and prognosis of BLCA patients.
