# Supplementary material for: Unveiling the role of YARS1 in bladder cancer: A prognostic biomarker and therapeutic target
Source: J Cell Mol Med. 2024 Mar 20;28(7):e18213. doi: 10.1111/jcmm.18213 (PMC10951887; doi:10.1111/jcmm.18213)
Supplement: Supplementary file 2 — Figure S2. [file JCMM-28-e18213-s001.docx]

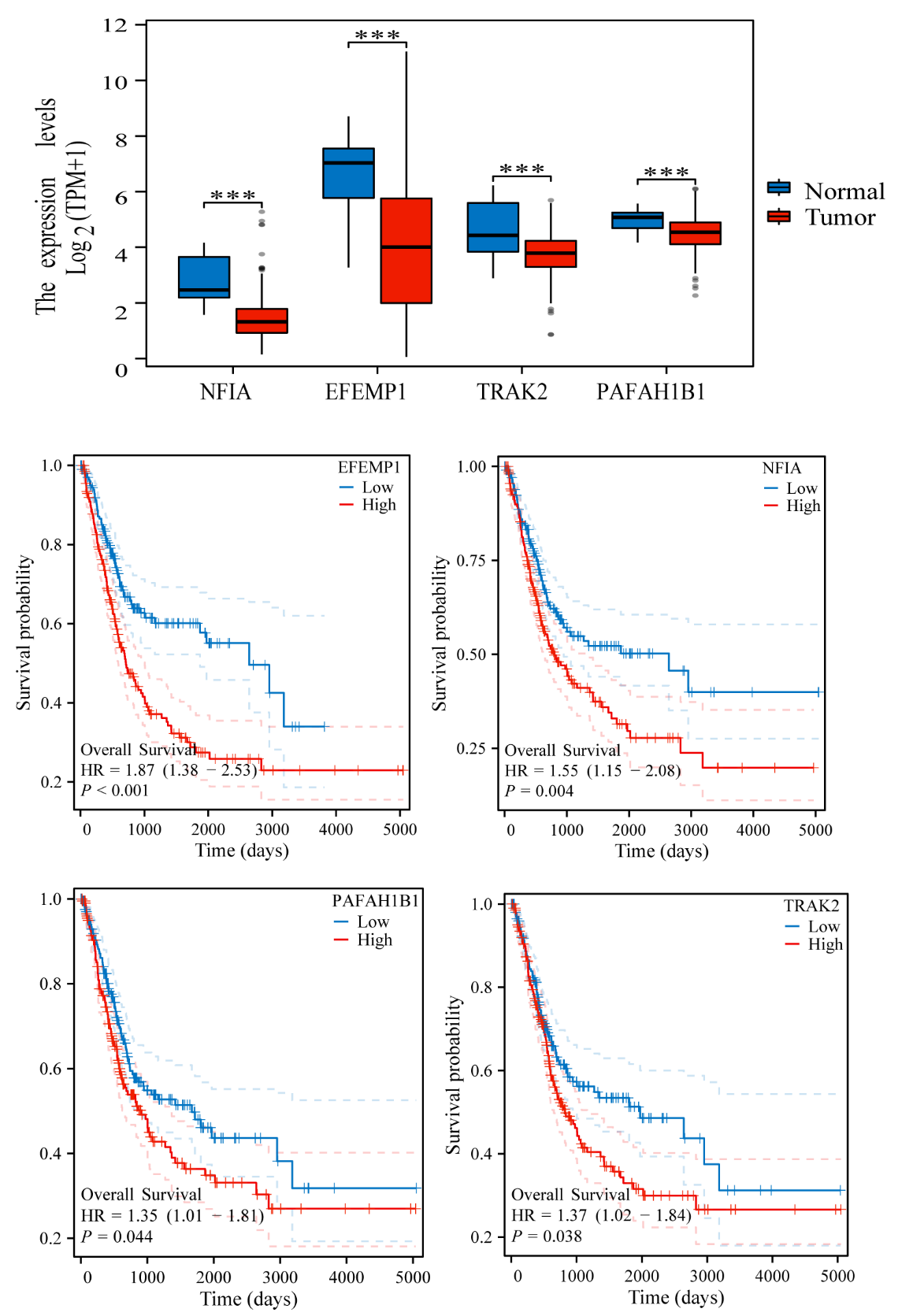


Supplementary figure 2 Expression and prognostic differences of four YARS1-related circRNAs in BLCA. (A) Expression of all four YARS1-related circRNAs was lower in BLCA than in normal bladder tissue. (B) All four YARS1-associated circRNAs were highly expressed in BLCA suggesting a poor prognosis for the patients.
